# 18β-glycyrrhetinic acid suppresses experimental autoimmune encephalomyelitis through inhibition of microglia activation and promotion of remyelination

**DOI:** 10.1038/srep13713

**Published:** 2015-09-02

**Authors:** Jieru Zhou, Wei Cai, Min Jin, Jingwei Xu, Yanan Wang, Yichuan Xiao, Li Hao, Bei Wang, Yanyun Zhang, Jie Han, Rui Huang

**Affiliations:** 1 Department of Rheumatology, East Hospital, Tongji University School of Medicine, Shanghai, China; 2 Medical College of Soochow University, Suzhou, Jiangsu, China; 3 Department of Infectious Diseases, Ruijin Hospital, Shanghai Jiao Tong University School of Medicine, Shanghai, China; 4 Key Laboratory of Stem Cell Biology, Institute of Health Sciences, Shanghai Institutes for Biological Sciences, Chinese Academy of Sciences and Shanghai Jiao Tong University School of Medicine (SJTUSM), and Shanghai Institute of Immunology, Institutes of Medical Sciences, SJTUSM, Shanghai, China; 5 Clinical Laboratory, the First Affiliated Hospital of Anhui Medical University, Hefei, China

## Abstract

Microglia are intrinsic immune cells in the central nervous system (CNS). The under controlled microglia activation plays important roles in inflammatory demyelination diseases, such as multiple sclerosis (MS). However, the means to modulate microglia activation as a therapeutic modality and the underlying mechanisms remain elusive. Here we show that administration of 18β-glycyrrhetinic acid (GRA), by using both preventive and therapeutic treatment protocols, significantly suppresses disease severity of experimental autoimmune encephalomyelitis (EAE) in C57BL/6 mice. The treatment effect of GRA on EAE is attributed to its regulatory effect on microglia. GRA-modulated microglia significantly decreased pro-inflammatory profile in the CNS through suppression of MAPK signal pathway. The ameliorated CNS pro-inflammatory profile prevented the recruitment of encephalitogenic T cells into the CNS, which alleviated inflammation-induced demyelination. In addition, GRA treatment promoted remyelination in the CNS of EAE mice. The induced remyelination can be mediated by the overcome of inflammation-induced blockade of brain-derived neurotrophic factor expression in microglia, as well as enhancing oligodendrocyte precursor cell proliferation. Collectively, our results demonstrate that GRA-modulated microglia suppresses EAE through inhibiting microglia activation-mediated CNS inflammation, and promoting neuroprotective effect of microglia, which represents a potential therapeutic strategy for MS and maybe other neuroinflammatory diseases associated with microglia activation.

Microglia are central nervous system (CNS)-specific macrophages that viewed as the major immunocompetent element in the CNS in charge of sensing any brain-damaging event^1^,^2^. Increasing studies suggest that activation of microglia is a hallmark of inflammatory demyelinating diseases such as multiple sclerosis (MS) and the animal model, experimental autoimmune encephalomyelitis (EAE)^3^,^4^. In MS and EAE, microglia exhibit uncontrolled activation, produce pro-inflammatory mediators, which recruit encephalitogenic T cells into the CNS, and play a leading role in oligodendrocyte death and demyelination^5^,^6^,^7^. However, when microglia activation is properly modulated, they can promote CNS remyelination through increased neurotrophic factor production^8^,^9^,^10^, which is in accordance with our recent results^11^. Therefore, the development of new therapeutic approaches designed to modulate activation of microglia, while preserving their neuroprotective effects, would suppress EAE pathogenesis and be great beneficial for MS therapy.

To this end, we employed such an approach to identify novel therapeutic compounds for EAE and to characterize the underlying regulatory mechanisms. We recently find that 18β-glycyrrhetinic acid (GRA), a chemically defined compound, shows a potent inhibitory effect on the inflammatory activation of liver-resident macrophages, Kupffer cells^12^. In addition, GRA exhibits neuroprotective effects^13^, which prompted us to examine whether GRA has potential regulatory effects on modulation of CNS-resident macrophages, microglia in EAE. Our data indicate that GRA effectively suppresses EAE disease severity, and the treatment effect is attributed to GRA-modulated microglia, which reduce the recruitment of encephalitogenic T cells in the CNS, as well as promote oligodendrocyte precursor cell (OPC)-mediated CNS remyelination.

## Results

### GRA reduces CNS inflammation and myelin damage in EAE

**Figure 1:**
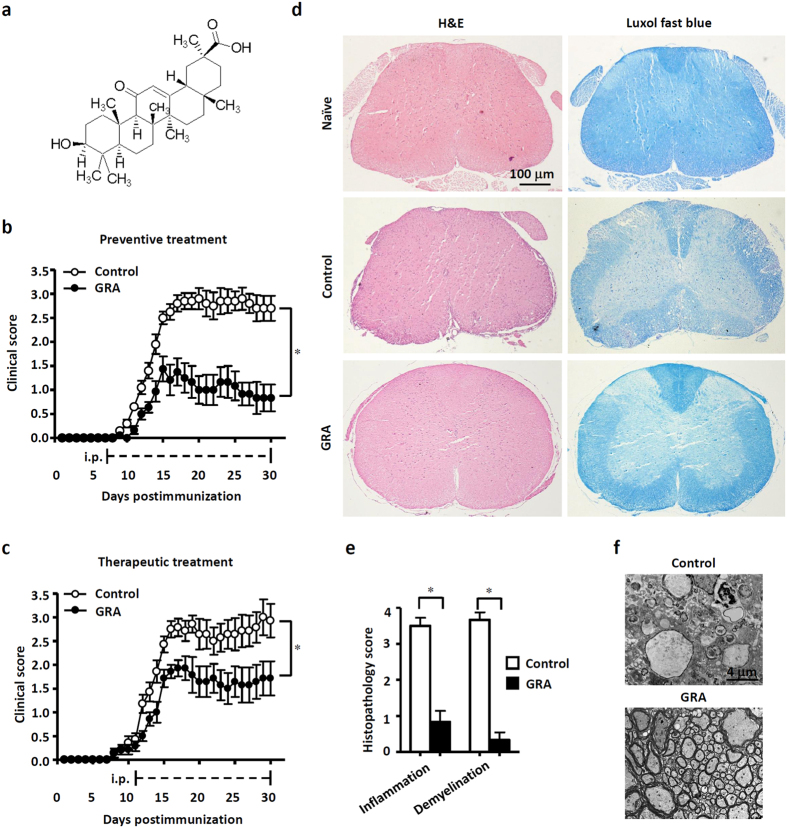
GRA reduces CNS inflammation and myelin damage in EAE. (**a**) Chemical structure of GRA. (**b**,**c**) Clinical scores of EAE mice that were i.p. injected daily with GRA (75 mg/kg) or vehicle control from day 7 (n = 10, preventive treatment) or day 11 (n = 7, therapeutic treatment)-post immunization onwards. (**d**) Transverse sections of spinal cords from control EAE mice and GRA-treated mice were stained with H&E or Luxol fast blue (×40). (**e**) Histopathology score of CNS inflammation and demyelination was quantified using H&E and Loxol fast blue staining on day 20 post immunization. (**f**) TEM images showed demyelinated axons in the spinal cords of control EAE and GRA-treated mice on day 25. Data are representative of three independent experiments. *P < 0.05.

To explore the efficacy of GRA, a natural pentacyclic triterpene (Fig. 1a) on actively induced EAE, GRA or vehicle control was administered i.p. daily in MOG_35–55_-immunized mice beginning on two different days: at day 7 as a preventive treatment protocol, when no clinical symptoms were observed; and at day 11 as a therapeutic treatment protocol. As illustrated in Fig. 1b and Fig. S1a when administered daily from day 7-post immunization onwards, GRA with the optimal dose 75 mg/kg showed a significant inhibitory effect on the severity of EAE as compared with the vehicle control. The clinical effects became overt at the time of disease onset (day 15) and persisted over the course of EAE. Similar efficacy was observed following the therapeutic treatment protocol (Fig. 1c). The effects of the treatment starting at day 15-post immunization was not as prominent as that of day 11-post immunization although mice attained clinical scores close to the maximum severity at day 15-post immunization (Fig. S1b).

To further differentiate pathological changes in EAE upon GRA treatment, we performed histologic analyses to examine CNS inflammatory infiltration and demyelination using the preventive treatment protocol. As shown in Fig. 1d,e, spinal cords from GRA-treated mice contained much lower incidences of both CNS inflammatory cell infiltration and demyelinated regions in the white matter compared with control EAE mice. In addition, transmission electron microscopy (TEM) showed that lower EAE scores did indeed reflect decreased myelin damage in GRA-treated mice (Fig. 1f). Taken together, these results indicate that GRA effectively reduces EAE severity by suppressing CNS inflammation and demyelination.

### GRA prevents the recruitment of encephalitogenic T cells into the CNS in EAE

**Figure 2:**
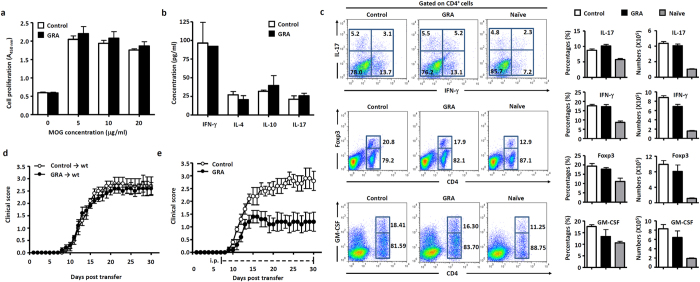
GRA prevents the recruitment of encephalitogenic T cells into the CNS in EAE. (**a**–**c**) Splenocytes from naïve, control EAE and GRA-treated mice at day 15 following the therapeutic treatment protocol were stimulated with MOG *ex vivo*, and proliferation of MOG-reactive T cells, cytokine profile and proportion of Th1, Th17 and Treg were analyzed. Representative dot plots were shown on the left, percentages and absolute numbers of cells were shown on the right (n = 6). (**d**) DLN cells and splenocytes from control EAE (n = 5) or GRA-treated mice (n = 5) at day 10 were transferred into sublethally irradiated mice. Mice were monitored and scored daily. (**e**) DLN cells and splenocytes from day 10 EAE mice were cultured with MOG and then transferred into sublethally irradiated mice. Recipients were treated with GRA (n = 5) or vehicle control (n = 5) from day 7 post transfer onwards. Mice were monitored and scored daily. Data are representative of three independent experiments.

To investigate the mechanisms by which *in vivo* administration of GRA attenuated disease severity of EAE, day 15 splenocytes were isolated from GRA-treated and control EAE mice and characterized for *ex vivo* T cell reactivity and cytokine profile in response to MOG challenge. The results revealed that the proliferation of MOG-reactive T cells derived from GRA-treated mice was similar to that of controls (Fig. 2a). In addition, there was no significant difference in the amounts of cytokines (IFN-γ, IL-4, IL-10 and IL-17) released by T cells obtained upon MOG stimulation of GRA-treated or control EAE mice (Fig. 2b). Moreover, GRA treatment did not alter the proportion and quantity of T helper (Th) 1 cells, Th17 and regulatory T cells (Treg) in EAE mice (Fig. 2c). Recent studies report that granulocyte-macrophage colony-stimulating factor (GM-CSF) in the encephalitogenicity of T cells is involved in EAE development^14^,^15^. As expected, GRA treatment did not alter the proportion and quantity of GM-CSF^+^ T cells in accordance to IFN-γ^+^ and IL-17^+^ T cells in EAE mice (Fig. 2c).

We next examined the ability of encephalitogenic T cells from GRA-treated and control MOG-immunized mice to confer EAE through adoptive transfer. Cells of draining lymph nodes (DLN) and splenocytes from GRA-treated or control EAE mice were adoptively transferred by i.v. injection into sublethally irradiated mice. As shown in Fig. 2d, mice from both groups developed typical EAE disease, with no significant difference in severity. These results indicate that the ameliorated EAE pathology conferred by GRA is not caused by an impaired encephalitogenic T cell response in the periphery.

The absence of an effect of GRA on the encephalitogenic T cell response prompted us to examine whether GRA affects the migration of encephalitogenic T cells into CNS. To examine this issue, activated MOG-specific T cells were obtained by coculture of MOG peptide with DLN cells and splenocytes from EAE mice. Passive EAE was established by transferring the activated MOG-specific T cells through i.v. injection into sublethally irradiated mice that were then treated i.p. with GRA or vehicle control. The result showed that GRA significantly inhibited the disease severity of passive EAE (Fig. 2e). This implies that GRA prevents the recruitment of encephalitogenic T cells into the CNS in EAE.

### GRA inhibits inflammatory infiltration through suppressed chemokine expression in the CNS of EAE mice

**Figure 3:**
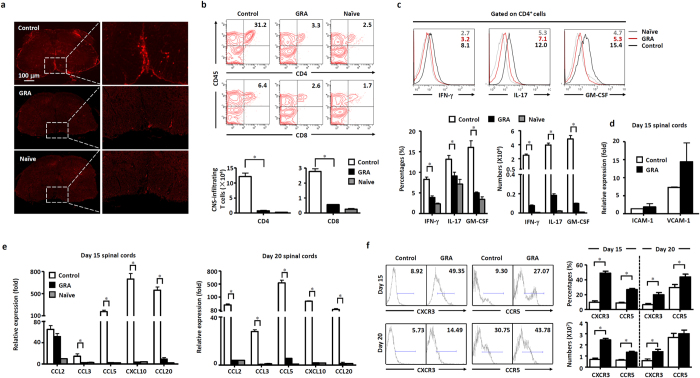
GRA inhibits chemokine expression and proinflammtory infiltration of T cells in the CNS of EAE mice. (**a**) Representative images of CD4 T cell infiltration in day 15 spinal cords of naïve, control and GRA-treated EAE mice following the therapeutic treatment protocol. (**b**) CNS-infiltrating T cells isolated from spinal cords from naïve, control EAE and GRA-treated mice at day 15 were analyzed. Representative dot plots were shown on the upper panel percentages and absolute numbers of cells are shown on the bottom panel (n = 6). (**c**) Spinal cords were isolated from naïve, control EAE and GRA-treated mice at day 15, surface marker and intracellular expression of cytokines were analyzed. Representative dot plots were shown on the upper panel, percentages and absolute numbers of cells were shown on the bottom panel (n = 6). (**d**) Quantification of mRNA abundance for ICAM-1 and VCAM-1 in the CNS from day 15 control and GRA-treated EAE mice. (**e**) Quantification of mRNA abundance for chemokines in the CNS from day 15 and 20 control and GRA-treated EAE mice. (**f**) The proportion of CXCR3^+^CD4^+^ and CCR5^+^CD4^+^ T cells in DLN cells from day 15 and 20 control and GRA-treated EAE mice. Representative dot plots were shown on the left, percentages and absolute numbers of cells were shown on the right (n = 6). Data are representative of three independent experiments. *P < 0.05.

We performed immunohistochemistry to detect CD4^+^ T cell infiltration in the CNS. As shown in Fig. 3A, in control EAE mice, there were significantly more CD4^+^ T cells in the spinal cords than that in GRA-treated EAE mice. In addition, the infiltration of encephalitogenic T cells (CD4^+^CD45^+^ and CD8^+^CD45^+^ T cells) in the CNS of GRA-treated mice were significantly decreased as compared with that in control EAE mice (Fig. 3b). Besides GRA treatment decreased the proportion and quantity of activated effector T cells in CNS (Fig. 3c). The above results indicated the impaired infiltration of encephalitogenic T cells into the CNS as a prominent feature under GRA treatment in EAE mice. The mechanisms underlying the CNS recruitment of encephalitogenic T cells depend on the release of chemotactic mediators and the expression of cell adhersion molecules on endothelial cells in the CNS. However, in this study, GRA showed no significant effect on the expression of adhersion molecules, such as ICAM-1 and VCAM-1 in the CNS of EAE mice^16^ (Fig. 3d).

Chemokines, including CCL2, CCL3, CCL5, CXCL10 and CCL20 play important roles in migration of immune cells into the CNS during EAE pathogenesis^17^. We found that the expression of CCL2, CCL3, CCL5, CXCL10 and CCL20 in the CNS reduced significantly in GRA-treated mice compared with control group among which the most conspicuous being CCL5 and CXCL10 (Fig. 3e). Accordingly, the proportion and absolute number of CXCR3^+^/CCR5^+^CD4^+^ encephalitogenic T cells were significantly higher in the DLN of day 15 and day 20 GRA-treated mice than in control EAE mice (Fig. 3f). These results indicate the migration of encephalitogenic T cells into the CNS was inhibited by GRA treatment through suppressed CNS chemokine expression.

### GRA inhibits microglia activation through suppression of MAPK signal

In EAE, activated microglia produce increased neurotoxic pro-inflammatory cytokines and chemokines that promote inflammatory infiltration in the CNS^18^,^19^. As GRA-treated mice present a normal immune response in the periphery, while decreased inflammatory profiles in the CNS, we hypothesized that the resistance of these mice to EAE pathology might be due to impaired pro-inflammatory activities in CNS-resident cells such as microglia or in infiltrating macrophage.

To characterize the cellular infiltration in the CNS of EAE mice and to determine which cell populations were altered by GRA treatment, we used chimeric mice that reconstituted with GFP bone marrow (Fig. S2) to distinguish between infiltrating macrophages (CD11b^+^GFP^+^) and CNS-resident microglia (CD11b^+^GFP^−^). To evaluate whether GRA treatment resulted in decreased recruitment of macrophages of peripheral origin, we detected the proportion of CNS-derived GFP^+^CD11b^+^ cells. The results showed that the proportion of CNS-derived GFP^+^CD11b^+^ cells has no difference between control and GRA-treated mice, but the absolute numbers of CNS-infiltrating GFP^+^CD11b^+^ peripheral macrophages are actually lower in GRA-treated mice as compared to control EAE mice. GRA treatment alleviated the autoimmune inflammation in the CNS, leading to dramatically decreased infiltration total mononuclear into CNS. However, GRA treatment did not alter the proportion and pattern of infiltrated mononuclear cell subpopulation (Fig. S3a). These data suggest that the therapeutic effect of GRA may related with the activated microglia but not infiltrating macrophages.

**Figure 4:**
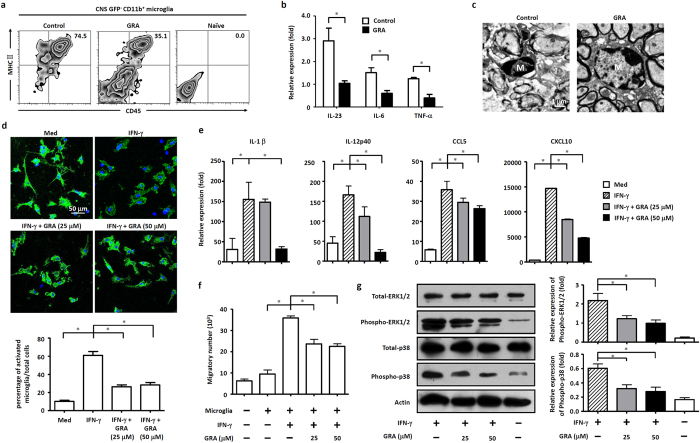
GRA inhibits microglia activation through suppression of MAPK signal pathway. (**a**) Flow cytometry analysis of activation markers (CD45 and MHC ΙΙ) on gated GFP^−^CD11b^+^ microglia isolated from the CNS of naïve, control and GRA treatment GFP-chimeric mice assessed on 15 day post immunization. Data are representative of four mice per group. (**b**) qPCR analysis of the indicated genes in FACS-sorted GPF^−^CD11b^+^ microglia isolated from naïve, control and GRA treatment GFP-chimeric EAE mice on 15 day post immunization. (**c**) TEM analyses of microglia status in spinal cords of day 15 control and GRA-treated EAE mice. (**d**) Representative images of primary microglia cultured with medium alone or treated with IFN-γ (100 ng/ml) or IFN-γ (100 ng/ml) plus GRA at the indicated concentrations, and the percentage of activated microglia with amoeboid shape normalized to total microglia (CD11b^+^). CD11b, green; DAPI, blue. *P < 0.05. (**e**) Primary microglia were cultured with medium alone or treated with IFN-γ (100 ng/ml) or IFN-γ (100 ng/ml) plus GRA at the indicated concentrations. Quantification of mRNA abundance for IL-1β, IL-12p40, CCL5 and CXCL10. (**f**) Culture supernatants were collected and cocultured with DLN cells isolated from day 15 EAE mice. The chemotactic ability was analyzed by counting the amount of migratory cells in the lower chamber. (**g**) Protein extracts from cultured microglia for 48 h were analyzed by western blot for expression of phosphorylated ERK1/2 and p38. Quantification results were shown on the right. Data are representative of three independent experiments. *P < 0.05.

We detected microglial activation depend on the acquisition of markers CD45 and MHC class ΙΙ (MHC ΙΙ)^20^. After EAE induction, control chimeras had a substantially increased frequency of activated microglia (CD45^+^ MHC ΙΙ^+^ GFP^−^) and a decreased frequency of resting microglia (CD45^−^ MHC ΙΙ^−^ GFP^−^), whereas the GRA treatment chimeras contained predominantly resting microglia (Fig. 4a), suggesting impaired microglia activation status in GRA-treated mice. Besides, microglia isolated from GRA treatment chimera mice, also had impaired expression of pro-inflammatory cytokines compared with the control (Fig. 4b). The active microglia presented long cisternae of granular endoplasmic reticulum and more irregular cell body shapes and exhibited phagocytosis of destroyed myelin and cellular debris. GRA treatment suppressed phagocytosis by microglia, as characterized by fewer fragments in the cytoplasm, suggesting impaired microglia activation (Fig. 4c). In control EAE mice, activated microglia with more irregular cell body shapes gather together in demyelinated regions, showing enhanced phagocytic ability in engulfment of damaged cells and cellular parts. By contrast, microglia were dispersed in GRA-treated mice, showing suppressed activation status. To confirm the inhibitory effect of GRA on microglia activation, primary cultured microglia with approximately 95% purity were prepared. Resting microglia presented a dendritic-like morphology with highly ramified processes (Fig. 4d). While activated with IFN-γ, microglia became amoeboid in shape. However, GRA addition reversed IFN-γ-induced microglia activation, and these cells exhibited ramified cytoplasmic processes of various degrees in microglia.

The inhibitory effect of GRA on IFN-γ-induced activation of microglia encouraged us to investigate the underlying mechanisms. We investigated the expression of several activation-associated, pro-inflammatory genes in primary microglia subjected to various treatments. Results revealed that the mRNA abundance for IL-1β, IL-12, CCL5 and CXCL10 was significantly up-regulated in IFN-γ-treated microglia. However, the up-regulation was inhibited by GRA addition to microglia cultures in a dose-dependent manner (Fig. 4e). In line with these findings, the migration of encephalitogenic T cells to the supernatants from IFN-γ induced GRA-treated primary microglia cultures were markedly inhibited as compared with that from IFN-γ-induced primary microglia cultures (Fig. 4f). However, GRA didn’t affect the activation of macrophage, or marginal effect (Fig. S3b,c). These results indicate that GRA suppresses CNS pro-inflammatory profile through inhibition of microglia activation during EAE pathogenesis.

The MAPK signaling transduction pathway plays an important role in IFN-γ-induced expression of pro-inflammatory genes in activated microglia^21^,^22^. Western blot analyses showed that IFN-γ induced marked elevation of phosphorylation of ERK1/2 and p38 in microglia. However, the elevation can be blocked by GRA in a dose-dependent manner (Fig. 4g). These results demonstrate that GRA decreases microglia production of pro-inflammatory cytokines and chemokines, and the inhibitory effect can be mediated by suppression of IFN-γ-induced activation via the MAPK signal pathway.

### GRA promotes remyelination in EAE

**Figure 5:**
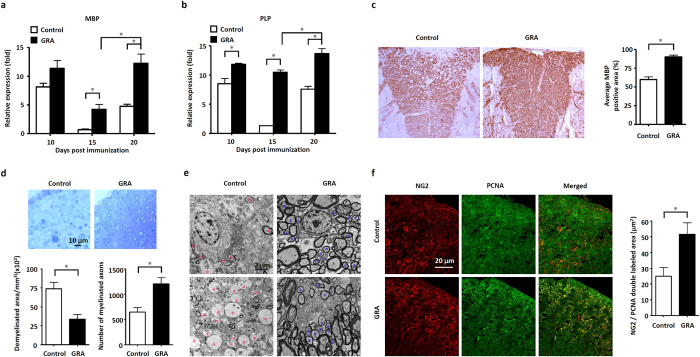
GRA promotes remyelination in EAE. (**a**,**b**) Quantification of mRNA abundance for MBP and PLP in the CNS from control and GRA-treated EAE mice at day 10, 15 and 20 following therapeutic treatment protocol (n = 6). (**c**) Representative images of MBP immunostaining in spinal cords from control EAE and GRA-treated mice at day 35, and quantification of MBP positively stained areas in spinal cords of GRA-treated and control group. (**d**) Toluidine blue staining of myelinated axons in day 30 spinal cords from control EAE and GRA-treated mice, and quantification of demyelinated areas and the number of myelinated axons in GRA-treated and control EAE mice using toluidine staining. (**e**) TEM showing demyelinated axons (red asterisks) in control spinal cords, and different remyelination phases with newly formed myelinated axons (blue asterisks) in spinal cords from GRA-treated day 30 EAE mice. (**f**) Spinal cords from day 30 GRA-treated and control EAE mice were stained with NG2 and PCNA for analyses of the proliferation of OPCs. Data are representative of three independent experiments. *P < 0.05.

The amelioration of EAE pathology results from two major aspects of function, the suppression of CNS inflammation and the promotion of remyelination^23^,^24^. We then examined whether GRA treatment played roles in CNS remyelination. Previous studies revealed that the expression of myelin genes, such as myelin basic protein (MBP) and proteolipid protein (PLP), increased during remyelination, and this early alteration in gene expression is believed to reflect an important step leading to the remyelination process^25^,^26^. As illustrated in Fig. 5a, the expression of MBP was almost at the same level at day 10 in the CNS of GRA-treated and control EAE mice. Although the expression of MBP decreased sharply both in GRA-treated and control group at day 15, MBP expression was significantly higher in GRA-treated mice than in control EAE mice. On day 20, the expression of MBP up-regulated in both groups, but could not return to the level of day 10 in control EAE mice, which indicated permanent damage in the CNS. By contrast, GRA treatment led to significant recovery of MBP expression at day 20. Likewise, similar trends in PLP expression were observed in control and GRA-treated mice (Fig. 5b). In addition, immunohistochemical analyses revealed significant up-regulated expression of MBP in spinal cords of day 35 GRA-treated mice (Fig. 5c). These results suggest that GRA may promote the remyelination process in EAE mice.

We then used toluidine blue staining and TEM to determine myelin status in EAE mice. On day 30 in the CNS of GRA-treated mice, large amounts of thinly myelinated axons were observed around demyelinated regions (Fig. 5d). Different remyelination phases were observed under TEM analyses. In some visual fields, oligodendrocytes (with rounded or oval profile and few visible processes) were seen in close contact with newly-formed thinly myelinated axons (Fig. 5e). The presence of thinly myelinated axons in demyelinated regions provides strong evidence of axon remyelination under GRA treatment^27^.

Remyelination occurs when there are sources of oligodendrocytes, among which most come from OPCs and the crucial first step in OPC-mediated remyelination involves OPC proliferation^23^,^28^. We studied the effect of GRA on the status of OPCs, which were identified by their early phenotypic marker NG2. As shown in Fig. 5F, the number of NG2^+^ OPCs in the white matter of the spinal cords was markedly increased in day 30 GRA-treated mice compared with that of control EAE mice. Meanwhile, OPC proliferation was evaluated by colocalization of NG2 and PCNA. Immunohistochemical analyses revealed that GRA significantly increased the number of NG2^+^ PCNA^+^ OPCs in the white matter compared to control EAE mice. These results indicate that GRA induced remyelination in the CNS can be mediated by promotion of OPC proliferation.

### GRA-modulated microglia promote brain-derived neurotrophic factor (BDNF) expression

**Figure 6:**
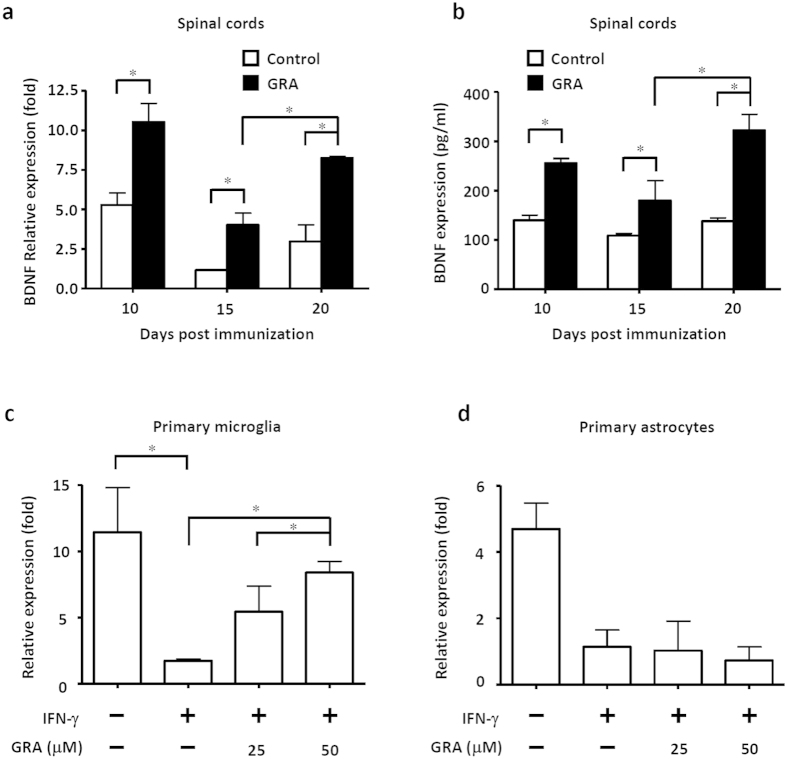
GRA-modulated microglia but not astrocyte exhibit promoted BDNF expression. (**a**) Quantification of mRNA abundance for BDNF in the CNS from control and GRA-treated EAE mice at day 10, 15 and 20 following therapeutic treatment protocol (n = 6). (**b**) Levels of serum BDNF in the CNS from control and GRA-treated EAE mice by ELISA at day 10, 15 and 20 (n = 6). (**c**) Primary microglia were cultured in medium alone or treated with IFN-γ (100 ng/ml) or IFN-γ (100 ng/ml) plus GRA at the indicated concentrations, and mRNA abundance for BDNF was quantified. (**d**) Primary astrocytes were cultured in medium alone or treated with IFN-γ (100 ng/ml) or IFN-γ (100 ng/ml) plus GRA at the indicated concentrations, and mRNA abundance for BDNF was quantified by real-time PCR. Data are representative of three independent experiments. *P < 0.05.

The neurotrophic factor BDNF plays a key role in neuronal and axonal survival, which is partly mediated by promoting OPC-mediated remyelination^29^. Deficiency of BDNF can lead to reduced NG2^+^ OPCs and decreased expression of MBP and PLP in the CNS^30^. We found similar trends of BDNF expression as MBP and PLP in the CNS of GRA-treated mice (Fig. 6a). The ELISA results showed similar trends of BDNF expression (Fig. 6b). As microglia are one of the major sources of BDNF in the CNS, we analyzed BDNF expression in primary microglia subjected to various treatments. Results showed that IFN-γ significantly decreased the expression of BDNF, whereas GRA blocked the down-regulation caused by IFN-γ (Fig. 6c). However, GRA failed to rescue BDNF down-regulation caused by IFN-γ in astrocytes (Fig. 6d), which are also the source of BDNF in the CNS. These results collectively indicate that GRA-induced promotion of OPC proliferation can be mediated by increased BDNF in the CNS.

## Discussion

In MS and EAE, microglia activate rapidly, produce large amounts of pro-inflammatory cytokines and chemokines, which mobilize more immune cells to infiltrate into the CNS, and ultimately result in oligodendrocyte death and demyelination^31^. Because microglia serve as the major immunocompetent element in the CNS and the first cells in the CNS to respond to neuronal damage, modulation of microglia function can be a therapeutic strategy in coping CNS inflammation and inflammation-induced demyelination. Here we demonstrate that GRA possesses unique therapeutic effects in EAE, which are mostly attributed to the modulatory effect of GRA on microglia, accompanied by significantly reduced CNS inflammation and myelin damage, as well as promotion of CNS remyelination in EAE mice.

Efficient activation of encephalitogenic T cells is an obligatory requirement for induction of CNS inflammation and pathology in EAE. While in this study, T cells derived from GRA-treated mice did not show any impaired activity as indicated by *in vitro* recall responses and adoptive transfer model, which further confirmed that GRA did not directly affect peripheral lymphocyte response^12^. However, adoptively transferred EAE mice exhibited suppressed clinical symptoms after treated with GRA, indicating that the alleviated EAE pathology in GRA-treated mice can be due to the prevented recruitment of encephalitogenic T cells into the CNS. Indeed, pro-inflammatory chemokines in the CNS were down-regulated, and the infiltration of inflammatory cells were reduced accordingly in GRA-treated mice. As the CNS-resident cells, such as microglia, are major producers of these chemokines in EAE^32^, we assumed that reduced chemokine production in the CNS by GRA may be mediated by modulation of microglia activation. This is proved by observation of the inhibited microglia activation and impaired expression of pro-inflammatory cytokine in the CNS and *in vitro* cultures after GRA treatment. However, as another group of important cells during EAE, the activation of macrophage wasn’t affected by GRA. GRA can affect cell functions by targeting gap junction channels^33^,^34^, toll-kike receptor^35^, cytochrome P450 2E1(CYP2E1)^36^ and so on. It has be regarded that these signalings such as gap junction channels paly critical roles in both microglia macrophages^37^. To explain the different effects of GRA on microglia and macrophages, further investigation is needed. Taken together, our data demonstrated that GRA showed potent ability in modulating microglia activation, leading to suppressed pro-inflammatory profile in the CNS of EAE mice. This alteration reduced the recruitment of encephalitogenic T cells into the CNS, and alleviated inflammation-induced demyelination in the CNS. Besides microglia were active in the induction phase of EAE, and the therapeutic effects of GRA is because of the inhibition of the microglia activation but not the encephalitogenic T cells. It may explain why day 15-post immunization when the mice developed severe clinical symptoms was not an optimal therapeutic time point.

The expression of myelin genes such as MBP and PLP in the CNS are considered to reflect the dynamic processes of demyelination and remyelination in EAE pathogenesis^25^,^26^. Our results demonstrated that GRA promoted MBP and PLP expression in the CNS during EAE process, together with the observation of the presence of thinly myelinated axons in demyelinated areas, indicating promoted remyelination in the CNS of EAE mice. Because the major source for remyelination in EAE depends on OPCs^23^,^28^, it is reasonable to assume that the promoted remyelination in GRA-treated mice results from the remyelinatory ability of OPCs^38^. Our data indicated that GRA treatment augmented the proliferation of OPCs in the white matter of the CNS. Moreover, the increased colocalization of NG2 and PCNA in the CNS provided strong evidence that GRA treatment had the ability to promote axon remyelination in the CNS in EAE mice.

Increasing studies as well as our previous work, had demonstrated that, properly modulated-microglia had the potential to promote OPC-mediated functional recovery in CNS inflammatory diseases^8^,^9^. BDNF is one of the major neurotrophic factors released by microglia, and was reported to promote OPC proliferation and the differentiation of oligodendrocyte^39^,^40^. In addition, BDNF deficiency impaired the proliferation and differentiation of OPCs, leading to reduced NG2^+^ OPCs and decreased expression of MBP and PLP in the CNS^30^. In EAE mice, the expression of BDNF decreased along with increased EAE severity. However, in GRA-treated mice, BDNF was significantly up-regulated in the CNS, with the expression trend similar to that of MBP and PLP. In addition, GRA overcame the blockade of BDNF expression caused by IFN-γ in primary microglia *in vitro*. These results indicated that the remyelination process of GRA-treated mice could be attributed to promoted BDNF expression by GRA-modulated microglia.

In conclusion, we demonstrated that GRA had a unique therapeutic potential for EAE through modulation of microglia activation via the inhibition of MAPK signal pathway. GRA-modulated microglia down-regulated CNS pro-inflammatory profile, which reduced the recruitment of encephalitogenic T cells into the CNS, and alleviated inflammation-induced demyelination. Importantly, GRA promoted remyelination through augmented OPC proliferation, which could be attributed to the increased BDNF in the CNS by GRA-modulated microglia. This study hopefully provides a new potential therapeutic approach to MS or maybe other neurological diseases based on modulation of microglia.

## Methods

### Ethics Statement

All animal experiments were performed in accordance with the guidelines from the Biomedical Research Ethics Committee of Shanghai Institutes for Biological Sciences, Chinese Academy of Sciences. The protocol used was reviewed and approved by the Animal Experiment Administration Committee of the Biomedical Research Ethics Committee of Shanghai Institutes for Biological Sciences, Chinese Academy of Sciences. All efforts were made to minimize the suffering of mice during experiments.

### Induction and treatment of EAE

Female C57BL/6 mice were purchased from Shanghai Laboratory Animal Center, Chinese Academy of Sciences, and were kept under pathogen-free condition in the animal center of the Shanghai Jiao Tong University School of Medicine (Shanghai, China). Mice (6–8 weeks of age) were immunized s.c. with the synthetic peptide (300 μg) MOG_35–55_ (MEVGWYRSPFSRVVHLYRNGK) (GL Biochem, Shanghai, China). Immunization was performed by mixing MOG peptide in CFA (Sigma-Aldrich, St. louis, MO, USA) containing 5 mg/ml *Mycobacterium tuberculosis* H37Ra (Difco Laboratories, Detroit, MI, USA). Pertussis toxin (200 ng; List Biological Laboratories, Campbell, CA, USA) in PBS was administered i.v. on days 0 and 2. For treatment of EAE, GRA (Sigma-Aldrich) or DMSO (Sigma-Aldrich) as vehicle control was administered at 75 mg/kg i.p. daily from day 7 or day 11 post-immunization onwards. For adoptively transferred EAE, splenocytes were harvested from EAE mice on day 10 post-immunization. The cells (5 × 10^6^ cells/ml) were cultured with MOG_35–55_ peptide (20 μg/ml) for 4 days and were then i.v. transferred into sublethally (500 rad) irradiated mice (2 × 10^7^ cells/mouse). The recipients received 200 ng of pertussis toxin by i.v. injection immediately after cell transferation and 2 days later. Recipients were also treated i.p. daily with GRA or vehicle control from day 7 of transfer onwards. In some experiments, mice were immunized s.c. with 300 μg of MOG_35–55_ emulsified in CFA and treated with GRA or vehicle control on the day of the first immunization. Ten days after immunization, cells from DLN (subiliac, proper axillary and accessory axillary)^41^ and spleens were isolated, pooled, and directly transferred into sublethally (500 rad) irradiated mice (2 × 10^7^ cells/mouse). The recipients received 200 ng of pertussis toxin by i.v. injection immediately after cell transfer and 2 days later. Mice were examined daily and scored for disease severity using the standard scale: 0, no clinical signs; 1, limp tail; 2, paraparesis (weakness, incomplete paralysis of one or two hind limbs); 3, paraplegia (complete paralysis of two hind limbs); 4, paraplegia with forelimb weakness or paralysis; 5, moribund or death. After the onset of EAE, food and water were provided on the cage floor. To eliminate any diagnostic bias, scores were assigned by researchers blinded to mouse identity. 50 mg/kg BrdU was injected intraperitoneally for 3 d before sacrificed to label dividing cells.

### Bone marrow chimeras

GFP-transgenic (C57BL/6-Tg (CAG-EGFP) 10sb/J) mice were purchased from from The Jackson Laboratory. We adoptively transferred lethally irradiated (950 rad) C57BL/6 mice (8 weeks of age) with mononuclear bone marrow cells (1 × 10^7^) derived from the GFP-transgenic mice. Under these conditions, the radioresistant CNS-resident cells would be retained, whereas the radiosensitive bone marrow and peripheral immune cells would be eliminated and replaced by the GFP^+^ cells. The bone marrow reconstitution does not affect the homeostasis of CNS-resident cells. We immunized the chimeric mice for EAE induction at 8 week after bone marrow reconstitution.

### Histopathology

Spinal cords from mice transcardially perfused with 4% (w/v) paraformaldehyde were dissected and postfixed overnight. Paraffin-embedded sections (8 μm) of spinal cords were stained with hematoxylin and eosin (H&E) or Luxol Fast Blue and examined by light microscopy (Nikon, Yurakucho, Tokyo, Japan). Semiquantitative analyses of inflammation and demyelination were performed in a blinded manner as previously described^11^.

### Cell proliferation and cytokine assay

Splenocytes isolated from MOG_35–55_-immunized mice were cultured in triplicate in complete DMEM at a density of 5 × 10^5^ per well in 96-well plates in the presence or absence of MOG peptide at the indicated concentration, and were maintained at 37 °C in 5% CO_2_ for 72 h. Cell proliferation was measured with CCK8 reagent (Dojindo Molecular Technologies Inc., Shanghai, China). For cytokine measurements, supernatants were collected from cell cultures at 48 h and diluted for the measurements of IFN-γ, IL-4, IL-10 and IL-17 by ELISA (R&D Systems, Minneapolis, MN, USA) according to the manufacturer’s instructions.

### Preparation of CNS mononuclear cells and flow cytometry

For preparation of CNS mononuclear cells, brains and spinal cords from MOG_35–55_-immunized mice were excised and dissociated for 45 min at 37 °C by digestion with collagenase IV (2 mg/ml, Sigmal-Aldrich) and DNase I (100 μg/ml, Sigmal-Aldrich) in DMEM medium. Dispersed cells were passed through a 70 μm nylon mesh and collected by centrifugation. CNS mononuclear cells were isolated through a Percoll density gradient and collected from the interface fraction between 37% and 70% Percoll. After extensive washing, suspensions of cells were stained with FITC-labeled anti-CD4, PE-conjugated anti-CD11b, APC-conjugated anti-CD45, PE-Cy7-conjugated anti-MHC ΙΙ, PE-Cy7-conjugated anti-CD8, PE-conjugated anti-CXCR3, PE-conjugated anti-CCR5 (all from BD Pharmingen, San Diego, CA, USA). Isotype controls were used for determination of negative cells. The stained cells were analyzed on a FACSAria instrument (BD Bioscience, San Diego, CA, USA). For Th1, Th17 and Treg cell analysis, cells were stained with surface marker, permeabilized with the Intracellular Fixation and Permeabilization Buffer Set (eBioscience, San Diego, CA,USA), and then stained with anti IFN-γ, anti-IL17A, GM-CSF, and anti-Foxp3 antibodies. All these antibodies were purchased from eBioscience, unless marked otherwise.

### Primary microglia culture and T cell coculture assay

Primary microglia cultures were prepared as previously described^11^. Briefly, cerebral cortical cells from newborn C57BL/6 mice were dissociated after a 30-min trypsinization (0.25%) and were plated in 75-cm^2^ culture flasks (Corning Inc., Corning, NY, USA) in DMEM supplemented with 10% heat-inactivated fetal calf serum, 100 U/ml penicillin, and 100 mg/ml streptomycin. The culture medium was changed after 24 h and then once in every 4 days. Two weeks later, microglia were obtained by mild trypsinization. Purified microglia comprised a cell population in which 95% stained positively with CD11b antibodies. For treatment assay, microglia were incubated with complete DMEM and stimulated with or without 100 ng/ml IFN-γ (R&D) in the presence or absence of GRA (25 μM and 50 μM) at 37 °C in a humidified incubator with 5% CO_2_. For cell migration assay, the isolated primary microglia that seeded in complete DMEM medium were stimulated with or without IFN-γ (100 ng/ml), and treated with different doses of GRA, 24 h later, the microglia culture supernatants were collected and added to the lower chambers of Transwell inserts (Millipore). DLN cells isolated from day 15 EAE mice were seeded into the upper chambers. The migration was carried out for 2 h. After incubation, the nonmigrated cells were removed from the upper surface of the filters, and the chemotactic ability was analyzed by counting the amount of migratory cells in the lower chamber.

### Quantitative real-time PCR

Total RNA was isolated using TRIzol (Invitrogen, Carlsbad, CA, USA) according to the manufacturer’s instructions, and reverse transcribed. mRNA abundance was determined by real-time PCR using SYBR Green master mix (Applied Biosystems, Foster City, CA, USA). Primer sequences are detailed in Table S1. Data were collected and quantitatively analyzed on an ABI Prism 7900 sequence detection system (Applied Biosystems). The mouse β-actin gene was used as an endogenous control for sample normalization.

### Western Blot

Protein extracted from cultured microglia subjected to various treatments were resolved in 12% SDS-polyacrylamide gels and transferred to PVDF membranes. The protein of interest was detected by immunostaining with rabbit anti-phospho-p38, p38, phospho-ERK1/2, ERK1/2 (all from Cell Signaling Technology, Danvers, MA, USA) or rabbit anti-actin (Sigma-Aldrich), followed by incubation with HRP-labeled secondary antibodies, and detection with chemiluminescence.

### Immunohistochemistry and immunocytochemistry

For immunohistochemistry staining, sections of spinal cords were incubated with rat anti-mouse CD11b (1:50, BD Pharmingen, San Diego, CA, USA), rabbit anti-mouse NG2 Ab (1:100, Chemicon International, USA & Canada), mouse anti-PCNA (1:5000 Abcam, Cambridge, UK,) or mouse anti-MBP (1:1000, Covance Inc., Princeton, New Jersey, USA), which were then labeled with FITC-conjugated goat anti-rat IgG (1:400, Santa Cruz, CA, USA), Cy3-conjugated goat anti-rabbit IgG (1:500, KPL, Kirkegaard & Perry Laboratories, Inc., Gaithersburg, Maryland, USA), Alexa Fluor 488 goat anti-mouse IgG (1:500, Invitrogen) or HRP-conjugated goat anti-mouse IgG (1:1000, KPL), and examined by confocal microscopy (Leica Camera AG, Wetzlar, Germany) or light microscopy (Nikon). For immunocytochemistry staining, microglia were fixed in 4% paraformaldehyde for 20 min, blocked in 1% BSA for 30 min, and incubated overnight with rat anti-mouse CD11b (1:50, BD Pharmingen). Subsequently, cells were incubated with FITC-conjugated goat anti-rat secondary antibody (1:400, Santa Cruz). Finally, the cells were counterstained with DAPI and examined under a confocal microscope (Leica).

### Toluidine blue staining and EM

Toluidine blue staining and EM analysis were performed as described previously (Xu *et al.*, 2013). Briefly, mice were anaesthetized and perfused with 0.1 M PBS followed by 0.1 M PBS containing 2.5% glutaraldehyde (pH 7.3). Lumbar spinal cords were sliced into 1mm sections. Thin sections were cut, stained with uranyl acetate and lead citrate and then analyzed under transmission EM (CM-120, Philips, Amsterdam, Netherlands).

### Statistical analysis

GraphPad Prism (version 4.0, GraphPad Software, Inc., La Jolla, CA, USA) and SPSS 17.0 software (SPSS Inc. Chicago, IL, USA) was used for statistical analyses. Significant differences were evaluated using an independent-samples *t* test or Wilcoxon rank test, except that multiple treatment groups were compared within individual experiments by ANOVA test. Kruskal-Wallis is used for EAE curves. Values of *P* less than 0.05 were considered significant. All values were presented as mean ± SEM.

## Additional Information

**How to cite this article**: Zhou, J. *et al.* 18β-glycyrrhetinic acid suppresses experimental autoimmune encephalomyelitis through inhibition of microglia activation and promotion of remyelination. *Sci. Rep.* **5**, 13713; doi: 10.1038/srep13713 (2015).

## Supplementary Material

Supplementary Information
