# Supplementary material for: 18β-glycyrrhetinic acid suppresses experimental autoimmune encephalomyelitis through inhibition of microglia activation and promotion of remyelination
Source: Sci Rep. 2015 Sep 2;5:13713. doi: 10.1038/srep13713 (PMC4557075; doi:10.1038/srep13713)
Supplement: Supplementary Information [file srep13713-s1.pdf]

Supplementary Information

18β-glycyrrhetinic acid suppresses experimental autoimmune encephalomyelitis through inhibition of microglia activation and promotion of remyelination

Jieru Zhou[1,2,#], Wei Cai[3,#], Min Jin[2,4,#], Jingwei Xu[4], Yanan Wang[4], Yichuan Xiao[4], Li Hao[5], Bei Wang[4], Yanyun Zhang[4], Jie Han[1], Rui Huang[2]

Supporting Materials and Methods

Bone marrow–derived macrophages (BMDM) were isolated by flushing the bone marrow from femurs and tibias of Female C57BL/6 mice (6–8 weeks) and then maintained in DMEM medium supplemented with 20% FBS and 20 ng/mL M-CSF. Five days later, adherent macrophages were dissociated and resuspended in DMEM supplemented with 0.5% FBS to starve overnight. For treatment assay, BMDM were stimulated with or without 100 ng/ml IFN-γ (R&D) in the presence or absence of GRA (25 μM and 50 μM) for 48 h at 37 °C in a humidified incubator with 5% CO2. For chemotactic experiments BMDM were seeded in 96-well plates, the BMDM culture supernatants were collected and cocultured with DLN cells isolated from day 15 EAE mice. The chemotactic ability was analyzed by counting the amount of migratory cells in the lower chamber.

Supporting Figure Legends

Supporting Fig.S1. Therapeutic effects of GRA in EAE. (A) Clinical scores of EAE mice that were i.p. injected with GRA (25, 75, 150 mg/kg) or vehicle control from day 7 (n = 10, preventive treatment)-post immunization onwards. Results are showed as mean ± SEM and representative of three independent experiment with similar results. (B). Clinical scores of EAE mice that were i.p. injected with GRA (75 mg/kg) or vehicle control from day 15 (n = 10, therapeutic treatment)-post immunization onwards. Data are representative of three independent experiments. * $P < 0.05$.

Supporting Fig.S2. Bone marrow reconstitution in chimeric mice. Flow cytometry analysis of GFP on gated CD4$^+$, CD8$^+$, CD11b$^+$, CD11c$^+$ cells isolated from the spleen in chimeric mice.

Supporting Fig. S3. GRA has no effects in the activation of macrophage. (A) Flow cytometry analysis of GPF$^+$CD11b$^+$ infiltrating macrophages in CNS isolated from naïve, control and GRA treatment GFP-chimeric EAE mice on 15 day post immunization. Data are representative of three mice per group. Representative dot plots were shown on the left, percentages and absolute numbers of cells were shown as mean ± SEM from three independent experiments on the right (n = 6). *$P < 0.05$. (B) BMDM were cultured with medium alone or treated with IFN-γ (100 ng/ml) or IFN-γ (100 ng/ml) plus GRA for 48 h at the indicated concentrations. Quantification

of mRNA abundance for IL-1β, IL-6, IL-10 and TNF-α. * $P < 0.05$. (C) Culture supernatants were collected and cocultured with DLN cells isolated from day 15 EAE mice. The chemotactic ability was analyzed by counting the amount of migratory cells in the lower chamber. * $P < 0.05$.

Figure S1

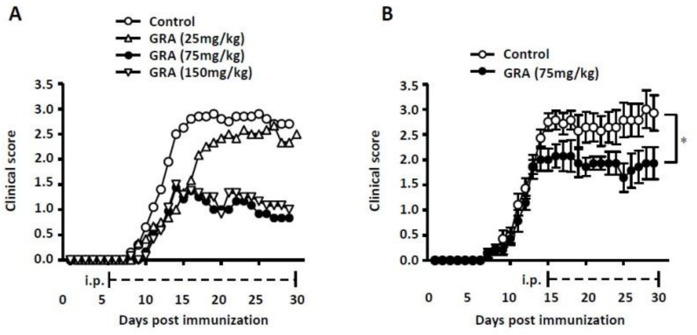

Figure S2

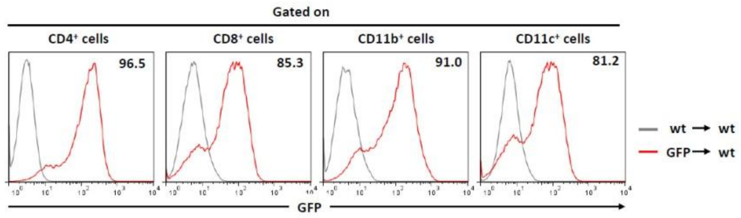

Figure S3

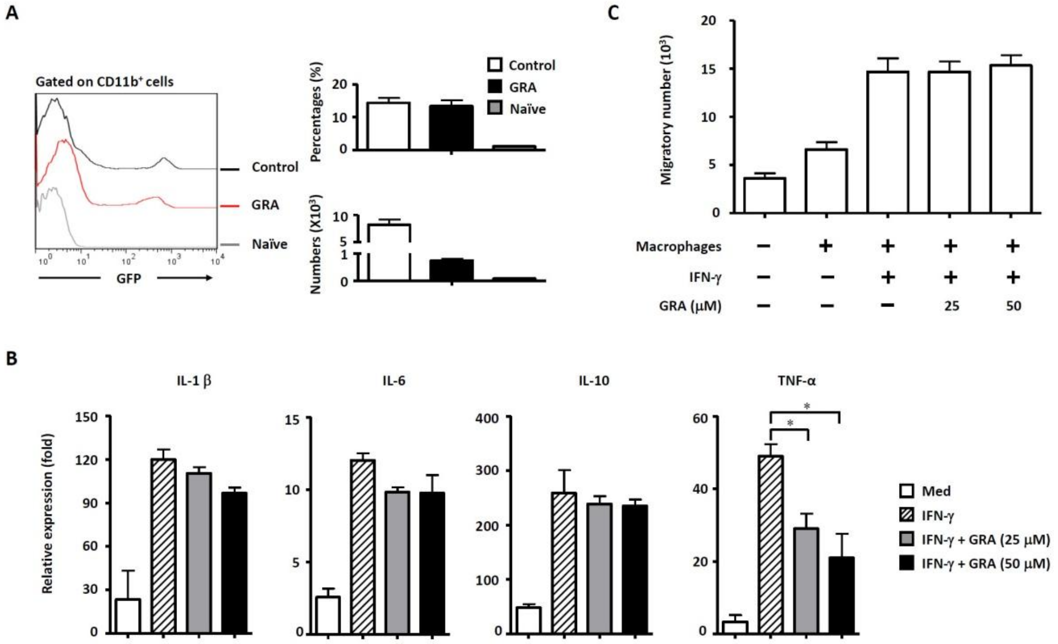

Table S1. Specific primers used in real-time PCR analysis

Gene	Primer	Sequence (5′→3′)
β-actin	FW	ATGGAGGGGAATACAGCCC
	RV	TTCTTTGCAGCTCCTTCGTT
CCL2	FW	ATTGGGATCATCTTGCTGGT
	RV	CCTGCTGTTCACAGTTGCC
CCL3	FW	ACCATGACACTCTGCAACCA
	RV	GTGGAATCTTCCGGCTGTAG
CCL5	FW	CCACTTCTTCTCTGGGTTGG
	RV	GTGCCCACGTCAAGGAGTAT
CXCL10	FW	CCTATGGCCCTCATTCTCAC
	RV	CTCATCCTGCTGGGTCTGAG
CCL20	FW	ACTGTTGCCTCTCGTACATACA
	RV	GAGGAGGTTCACAGCCCTTTT
IL-23	FW	TCCCTACTAGGACTCAGCCAACTC
	RV	ACTCAGGCTGGGCACTG
IL-6	FW	CACAGAGGATACCACTCCCAACA
	RV	TCCACGATTTCCCAGAGAACA
TNF-α	FW	CATCTTCTCAAAATTCGAGTGACAA
	RV	CCAGCTGCTCCTCCACTTG
IL-1β	FW	GGTCAAAGGTTTGGAAGCAG
	RV	TGTGAAATGCCACCTTTTGA
IL-12p40	FW	GGAAGCACGGCAGCAGAATA
	RV	AACTTGAGGGAGAAGTAGGAATGG
MBP	FW	GCTCCCTGCCCCAGAAGT
	RV	TGTCACAATGTTCTTGAAGAAATGG
PLP	FW	GCCCCTACCAGACATCTAGC
	RV	AGTCAGCCGCAAAACAGACT
BDNF	FW	GCCTTCATGCAACCGAAGTA
	RV	TGAGTCTCCAGGACAGCAAA
